# Neoadjuvant Radiochemotherapy Significantly Alters the Phenotype of Plasmacytoid Dendritic Cells and 6-Sulfo LacNAc^+^ Monocytes in Rectal Cancer

**DOI:** 10.3389/fimmu.2019.00602

**Published:** 2019-03-29

**Authors:** Felix Wagner, Ulrike Hölig, Friederike Wilczkowski, Ioana Plesca, Ulrich Sommer, Rebekka Wehner, Maximilian Kießler, Armin Jarosch, Katharina Flecke, Maia Arsova, Antje Tunger, Andreas Bogner, Christoph Reißfelder, Jürgen Weitz, Knut Schäkel, Esther G. C. Troost, Mechthild Krause, Gunnar Folprecht, Martin Bornhäuser, Michael P. Bachmann, Daniela Aust, Gustavo Baretton, Marc Schmitz

**Affiliations:** ^1^Institute of Immunology, Faculty of Medicine Carl Gustav Carus, Technische Universität Dresden, Dresden, Germany; ^2^Department of Radiotherapy and Radiation Oncology, University Hospital Carl Gustav Carus, Technische Universität Dresden, Dresden, Germany; ^3^Institute of Pathology, University Hospital of Dresden, Dresden, Germany; ^4^Partner Site Dresden, National Center for Tumor Diseases (NCT), Dresden, Germany; ^5^Partner Site Dresden, German Cancer Consortium (DKTK), and German Cancer Research Center (DKFZ), Heidelberg, Germany; ^6^Department of Medicine I, University Hospital Carl Gustav Carus, Technische Universität Dresden, Dresden, Germany; ^7^Department of Gastrointestinal, Thoracic, and Vascular Surgery, University Hospital Carl Gustav Carus, Technische Universität Dresden, Dresden, Germany; ^8^Department of Surgery, Mannheim University Medical Centre, University of Heidelberg, Mannheim, Germany; ^9^Department of Dermatology, University Hospital of Heidelberg, Heidelberg, Germany; ^10^OncoRay – National Center for Radiation Research in Oncology, Dresden, Germany; ^11^Institute of Radiooncology – OncoRay, Helmholtz-Zentrum Dresden-Rossendorf, Dresden, Germany; ^12^Department of Radioimmunology, Institute of Radiopharmaceutical Cancer Research, Helmholtz Center Dresden-Rossendorf, Dresden, Germany

**Keywords:** plasmacytoid dendritic cells, 6-sulfo LacNAc^+^ monocytes, CD8^+^ T cells, tumor immune architecture, radiochemotherapy, rectal cancer

## Abstract

Neoadjuvant radiochemotherapy (nRCT) can significantly influence the tumor immune architecture that plays a pivotal role in regulating tumor growth. Whereas, various studies have investigated the effect of nRCT on tumor-infiltrating T cells, little is known about its impact on the frequency and activation status of human dendritic cells (DCs). Plasmacytoid DCs (pDCs) essentially contribute to the regulation of innate and adaptive immunity and may profoundly influence tumor progression. Recent studies have revealed that higher pDC numbers are associated with poor prognosis in cancer patients. 6-sulfo LacNAc-expressing monocytes (slanMo) represent a particular proinflammatory subset of human non-classical blood monocytes that can differentiate into DCs. Recently, we have reported that activated slanMo produce various proinflammatory cytokines and efficiently stimulate natural killer cells and T lymphocytes. slanMo were also shown to accumulate in clear cell renal cell carcinoma (ccRCC) and in metastatic lymph nodes from cancer patients. Here, we investigated the influence of nRCT on the frequency of rectal cancer-infiltrating pDCs and slanMo. When evaluating rectal cancer tissues obtained from patients after nRCT, a significantly higher density of pDCs in comparison to pre-nRCT tissue samples was found. In contrast, the density of slanMo was not significantly altered by nRCT. Further studies revealed that nRCT significantly enhances the proportion of rectal cancer-infiltrating CD8^+^ T cells expressing the cytotoxic effector molecule granzyme B. When exploring the impact of nRCT on the phenotype of rectal cancer-infiltrating pDCs and slanMo, we observed that nRCT markedly enhances the percentage of inducible nitric oxide synthase (iNOS)- or tumor necrosis factor (TNF) alpha-producing slanMo. Furthermore, nRCT significantly increased the percentage of mature CD83^+^ pDCs in rectal cancer tissues. Moreover, the proportion of pDCs locally expressing interferon-alpha, which plays a major role in antitumor immunity, was significantly higher in post-nRCT tissues compared to pre-nRCT tumor specimens. These novel findings indicate that nRCT significantly influences the frequency and/or phenotype of pDCs, slanMo, and CD8^+^ T cells, which may influence the clinical response of rectal cancer patients to nRCT.

## Introduction

Colorectal cancer is one of the most common malignancies in the United States with an estimated incidence of 140,250 cases and an estimated number of 50,630 deaths in 2018 (1). Previous reports have provided evidence that the immune contexture plays a major role for the clinical outcome of colorectal cancer patients (2). Thus, it has been shown that high densities of CD45RO^+^ T helper (Th) 1 cells and CD8^+^ T cells are associated with improved survival of colorectal cancer patients (3, 4). However, patients with high expression of Th17 genes had a poor prognosis. These results led to the development of a so-called “immunoscore” for an optimized tumor classification (5).

Neoadjuvant radiochemotherapy (nRCT) followed by total mesorectal excision constitutes the current standard of care for locally advanced rectal cancers (6, 7). nRCT can efficiently reduce tumor size, resulting in a higher rate of sphincter-preserving surgical interventions, and an increased rate of R0-resections. In addition, this treatment modality decreases the local recurrence rate. Recent findings have revealed that nRCT can significantly influence the tumor immune contexture, affecting the tumor responsiveness to this treatment modality (2, 8–11). Thus, it has been reported that several chemotherapeutic agents as well as radiotherapy can efficiently stimulate antitumor immune responses by triggering immunogenic cell death in tumor cells (12, 13). This process is characterized by the translocation of intracellular calreticulin to the surface of tumor cells and the release of high-mobility-group box 1. Surface-exposed calreticulin markedly enhances the phagocytosis of tumor cells by dendritic cells (DCs) that play a crucial role in the induction and regulation of antitumor immunity (14). Released high-mobility-group box 1 from chemo- or radiotherapy-treated tumor cells promote the maturation and activation of DCs, resulting in an efficient processing and presentation of tumor-associated antigens and the stimulation of potent tumor-directed T-cell responses. In addition to immunogenic cell death induction, radiotherapy has been shown to reduce the surface expression of CD47, which acts as an antiphagocytosis signal to promote immune evasion (15). Inhibition of CD47 function significantly augments the engulfment of tumor cells by DCs, resulting in effective antitumor responses (16). In contrast to these immunostimulatory effects, radiotherapy, and chemotherapeutic agents can also induce immunosuppressive effects. They include the increase of tumor-infiltrating regulatory T cells and myeloid-derived suppressor cells, which produce immunosuppressive molecules (8, 9, 17). These immune cell subsets can profoundly impair the functional properties of effector T cells and can promote tumor growth and resistance.

Plasmacytoid DCs (pDCs) represent an important subset of human blood DCs that are capable of producing large amounts of interferon (IFN)-α upon stimulation (18, 19). In addition, pDCs can efficiently enhance the antitumoral capabilities of natural killer (NK) cells and T lymphocytes (20, 21). Based on these functional properties, stimulated pDCs can promote antitumor responses *in vivo*. Thus, the intratumoral administration of activated pDCs led to tumor regression in a B16 melanoma mouse model (22). Furthermore, it has been demonstrated in an orthotopic murine mammary tumor model that the intratumoral application of a toll-like receptor 7 ligand results in the activation of tumor-associated pDCs and tumor regression (23). In a clinical trial, intranodal injections of activated pDCs loaded with tumor-associated antigen-derived peptides in patients with metastatic melanoma induced specific CD8^+^ and CD4^+^ T cell responses (24). However, pDCs can also act as tolerogenic cells by suppressing T cell responses (19, 25). Previous studies have shown that pDCs infiltrate a variety of human cancers including head and neck, breast and ovarian cancer, and that a higher density is associated with poor clinical outcome (26–28). In addition, it has been demonstrated that tumor-infiltrating pDCs produce reduced amounts of IFN-α upon activation and can efficiently promote the expansion of regulatory T cells (29). 6-sulfo LacNAc (slan) monocytes (slanMo, formerly termed M-DC8^+^ DCs or slanDCs) are a subset of human non-classical blood monocytes, which can differentiate into DCs (30–40). Previously, we have reported that slanMo produce high levels of various proinflammatory cytokines and display a marked capability to handle IgG-complexed antigens (31, 32, 37). We have also demonstrated that slanMo mediate direct cytotoxicity against tumor cells (38, 39). Further studies have revealed that they efficiently induce neoantigen-specific CD4^+^ T cells, activate tumor-reactive CD8^+^ T cells, and promote the polarization of naïve CD4^+^ T lymphocytes into Th1 or Th17/Th1 cells (30–32, 36). Moreover, slanMo have been shown to stimulate IFN-γ production and cytotoxic activity of NK cells (39, 40).

In the present study, we determined the impact of nRCT on the frequency of rectal cancer-infiltrating pDCs, slanMo, CD3^+^ T cells, total CD8^+^ T lymphocytes, and GrzB-expressing CD8^+^ T cells. Furthermore, we analyzed the impact of nRCT on the percentage of rectal cancer-associated slanMo locally producing inducible nitric oxide synthase (iNOS) or tumor necrosis factor (TNF)-α, which play an important role in regulating tumor growth. Following recent findings, indicating that IFN-α essentially contributes to the antitumor effects mediated by RCT, the influence of nRCT on the proportion of rectal cancer-infiltrating pDCs locally expressing this cytokine was evaluated.

## Materials and Methods

### Patients and Study Design

This is a retrospective study including 60 rectal cancer patients treated with nRCT followed by surgery at the University Hospital Carl Gustav Carus of Dresden between 2001 and 2013. From 20 of these patients, tumor biopsies prior to nRCT were available. Additionally, a cohort of 28 primarily resected rectal cancer patients without nRCT was matched according to gender, age, and TNM-stage. Table 1 summarizes the clinicopathological characteristics of the study population.

**Table 1:** Clinicopathological characteristics of the rectal cancer patients.

	**nRCTx (*****n*** **=** **40)**		**Therapy-naïve (*****n*** **=** **28)**		**Matched group (*****n*** **=** **20)**	
	**Patients (*n*)**	**%**	**Patients (*n*)**	**%**	**Patients (*n*)**	**%**
**AGE**						
Median (range)	61.1 years (44.1–78.2)		64.5 years (22.1–76.5)		59.5 years (40.7–72.6)	
**GENDER**						
Male	31	77.5	19	67.9	13	65.0
Female	9	22.5	9	32.1	7	35.0
**pT**						
1	2	5.0	3	10.7	0	0.0
2	9	22.5	9	32.1	6	30.0
3a	14	35.0	10	35.7	9	45.0
3b	12	30.0	6	21.4	4	20.0
4	3	7.5	0	0.0	1	5.0
**pN**						
0	25	62.5	20	71.4	17	85.0
1	9	22.5	8	28.6	1	5.0
2	6	15.0	0	0.0	2	10.0
**nCT**						
5-FU	30	75.0			14	70.0
5-FU + Oxaliplatin	6	15.0			2	10.0
Others	4	10.0			4	20.0
**nRCT**						
55.8 Gy	1	2.5			2	10.0
50.4 Gy	39	97.5			18	90.0

### Immunohistochemistry

Formalin-fixed and paraffin-embedded tissue sections were cut into 3–5 μm sections. Subsequently, these sections were deparaffinized in xylene (2 × 15 min, VWR International, Fontenay-sous-Bois, France) and hydrated by washes of graded ethanol (Berkel AHK, Ludwigshafen, Germany) to water (B. Braun, Melsungen, Germany). Tissue sections were boiled in citrate buffer (Zytomed Systems GmbH, Berlin, Germany) at pH 6.0 for 20 min for antigen retrieval. Subsequently, tissues were stained overnight at 4°C with either the polyclonal goat anti-BDCA-2 antibody (1:200, R&D Systems, Minneapolis, MN, USA) to evaluate pDCs (41) or the monoclonal mouse anti-slan antibody DD2 (1:10, Institute of Immunology, Faculty of Medicine Carl Gustav Carus, Technische Universität Dresden, Dresden, Germany) to analyze slanMo (32, 34–36). Then, tissues used for pDC staining were incubated with a mouse anti-goat antibody solution (Thermo Fisher Scientific, Rockford, IL, USA) for 60 min. Afterwards, all tissues were incubated with dextran-labeled antibodies against mouse immunoglobulins (Dako, Glostrup, Denmark) for 30 min. pDCs and slanMo were visualized by the alkaline phosphatase-based EnVision^TM^ detection system according to the manufacturer's instructions (Dako). All tissue sections were counterstained with Mayer's hematoxylin (Merck, Darmstadt, Germany). For pDC quantification, positively stained cells were counted in three different high power fields (HPF) of a section by using AxioVision 4.8.1.0 (Zeiss, Jena, Germany) and the mean value was determined. The mean number of pDCs per HPF (area: 0.237 mm^2^) was converted to square millimeter. For slanMo, slides were digitized by an iScan Coreo slide scanner (Ventana Medical Systems, Oro Valley, AZ, USA) and evaluated using the same HPF method.

To determine the frequency of rectal cancer-infiltrating CD3^+^ T cells, CD8^+^ T cells, and granzyme B (GrzB)-expressing CD8^+^ T cells, formalin-fixed, and paraffin-embedded tissue sections were deparaffinized in xylene BenchMark XT (Ventana Medical Systems) and then exposed to the Cell Conditioning 1 solution for antigen retrieval (Ventana Medical Systems). Two double immunohistochemical stainings were performed: CD3 / Ki67 and CD8 / GrzB. For the first double reaction, the monoclonal mouse anti-CD3 antibody (clone 2GV6, ready-to-use, Ventana Medical Systems) and the monoclonal mouse anti-Ki67 antibody (clone Mib-1, 1:50, Dako) were used. For the second double staining, the monoclonal mouse anti-CD8 antibody (clone C8/144B, 1:10, Dako) and the monoclonal mouse anti-GrzB antibody (clone GrzB-7, 1:10, Dako) were applied. All tissue sections were counterstained with Mayer's hematoxylin. Subsequently, the tissue sections were digitized by an iScan Coreo slide scanner, followed by T-cell quantification by using the Image viewer v. 3.1 (Ventana Medical Systems). Positively stained T lymphocytes were counted in three different HPF of a section and the mean value was determined. The mean number of T cells per HPF (area: 0.237 mm^2^) was converted to square millimeter. To determine the percentage of GrzB-expressing CD8^+^ T lymphocytes, between 65 and 576 CD8^+^ T cells per tissue section were evaluated dependent on their frequency in the three HPF.

### Immunofluorescence Staining

Tissues were deparaffinized, hydrated, and heat-treated as described above. After antigen retrieval, tissue sections were incubated overnight with primary antibody solutions containing goat anti-human BDCA-2 (1:50, R&D Systems) and either mouse anti-human CD83 (clone 1H4b, 1:100, Abcam, Cambridge, UK) or mouse anti-human IFN-α (clone F-7, 1:500, Santa Cruz Biotechnology, Heidelberg, Germany). Subsequently, tissue sections were incubated with a rabbit anti-goat antibody solution (1:100, Abcam) for 10 min. Afterwards, secondary antibody solution containing donkey anti-rabbit AF488 (1:100, Abcam) and donkey anti-mouse AF546 (1:100, Thermo Fisher Scientific) was applied for 30 min.

For staining of slanMo, tissue sections were incubated overnight with primary antibody solutions containing rabbit anti-human TNF-α (1:100, Abcam) and mouse anti-human DD2 (1:20), followed by 30 min of incubation with the secondary antibodies goat anti-mouse IgM biotin (1:100, SouthernBiotech, Birmingham, AL, USA) and fluorescence-labeled goat anti-rabbit IgG AF488 (1:100, Thermo Fisher Scientific). Finally, Streptavidin AF546 (1:500, Thermo Fisher Scientific) was applied for 15 min. To determine iNOS expression in rectal cancer-infiltrating slanMo, tissue sections were incubated with a mouse anti-human iNOS antibody (1:50, BD Biosciences, San Jose, CA, USA) for 60 min, followed by the application of a goat anti-mouse IgG (1:400, Abcam) and fluorescence-labeled donkey anti-goat AF488 antibody (1:100, Thermo Fisher Scientific), each for 20 min. Mouse serum (1:100, Dako) was applied for 10 min to prevent unspecific binding of the following antibody. For the detection of slanMo, mouse anti-human DD2 antibody (1:2) was applied for 60 min. Afterwards the tissues were incubated with a secondary antibody solution containing goat anti-mouse IgM Biotin (1:100, SouthernBiotech), followed by the application of fluorophore AF546 labeled Streptavidin (1:500, Thermo Fisher Scientific), each for 20 min. Then, tissues were mounted with 4,6 diamidino-2-phenylindole-containing AKLIDES® ANA plus medium (Medipan, Dahlewitz/Berlin, Germany), coverslipped, and analyzed with a Keyence fluorescence microscope BZ-9000 (Keyence, Osaka, Japan). To determine the percentage of CD83^+^ and IFN-α^+^ pDCs or iNOS^+^ and TNF-α^+^ slanMo, between 20 and 50 cells per tissue section were evaluated dependent on their frequency.

For additional experiments, immunofluorescence multiplex staining was accomplished by using the Opal kit and the Vectra imaging platform (Perkin Elmer, Hopkinton, MA, USA). Tissues were deparaffinized and hydrated as described above. Antigen retrieval was performed in AR6 or AR9 buffer (both from PerkinElmer) using microwave treatment. Afterwards, the Opal kit was used according to the manufacturer's instructions (PerkinElmer). Therefore, tissue sections were blocked for 10 min with the Antibody Dilutent/Block (PerkinElmer), then incubated with the primary antibody for one hour, followed by 10 min incubation with a horseradish peroxidase-conjugated secondary antibody (PerkinElmer). In case of goat anti-human primary antibodies, another 10 min incubation with a bridge mouse anti-goat antibody (1:100, Thermo Fisher Scientific) was required prior to the application of the secondary antibody. Finally, a TSA fluorophore was added to the tissue sections for 10 minutes. Subsequent stripping of the primary together with secondary antibodies was performed by microwave treatment. In between all the steps mentioned above, except prior to the primary antibody application, tissue sections were washed for 2 x 3 min in TBST buffer. Every incubation step took place in a humidified chamber on a rocking platform at room temperature. Blocking, incubation with primary and secondary antibody, visualization by a TSA fluorophore and microwave treatment were repeated for each primary antibody. Finally, all tissue slides were counterstained with spectral DAPI (PerkinElmer) for 5 min, washed with TBST buffer and with autoclaved water for 2 min, and then coverslipped with fluoromount medium (SouthernBiotech).

For the four-color Opal multiplex staining, primary antibodies directed against CD8 (clone C8/144B, 1:100, Dako, high pH retrieval), BDCA-2 (goat polyclonal, 1:100, R&D Systems, low pH retrieval), and panCK (clone AE1/AE3, 1:100, Thermo Fisher Scientific, low pH retrieval) were used and visualized by the TSA fluorophores 570 (1:100), 650 (1:100), and 690 (1:100, all from PerkinElmer), respectively. For the two-color Opal staining, the BDCA-2 primary antibody was used together with the polyclonal goat anti-human CXCL10, CCL4, or CCL5 primary antibody (1:100, all from R&D Systems, low pH retrieval) and combined with the TSA fluorophores 650 (1:100) and 570 (1:100), respectively. Acquisition of the multispectral images was performed with the Vectra 3.5 Automated Imaging System (Perkin Elmer). Spectral unmixing was done in inForm^®^ using a library built from single stained tissue slides for each primary antibody-TSA fluorophore combination. ImageJ software was then used for final image processing.

### Statistical Analysis

Statistical analysis was performed using unpaired student's *t*-test for the evaluation of pDCs and slanMo in non-matched tissues of untreated and nRCT-treated rectal cancer patients. Paired student's *t*-test was used for the analysis of pDCs, slanMo, and T cells in matched pre-nRCT and post-nRCT tumor tissues. Values of ^*^p ≤ 0.05; ^**^p ≤ 0.01; ^***^p ≤ 0.001 were considered as significant.

## Results

### nRCT Significantly Increases the Frequency of pDCs in Rectal Cancer

**Figure 1:**
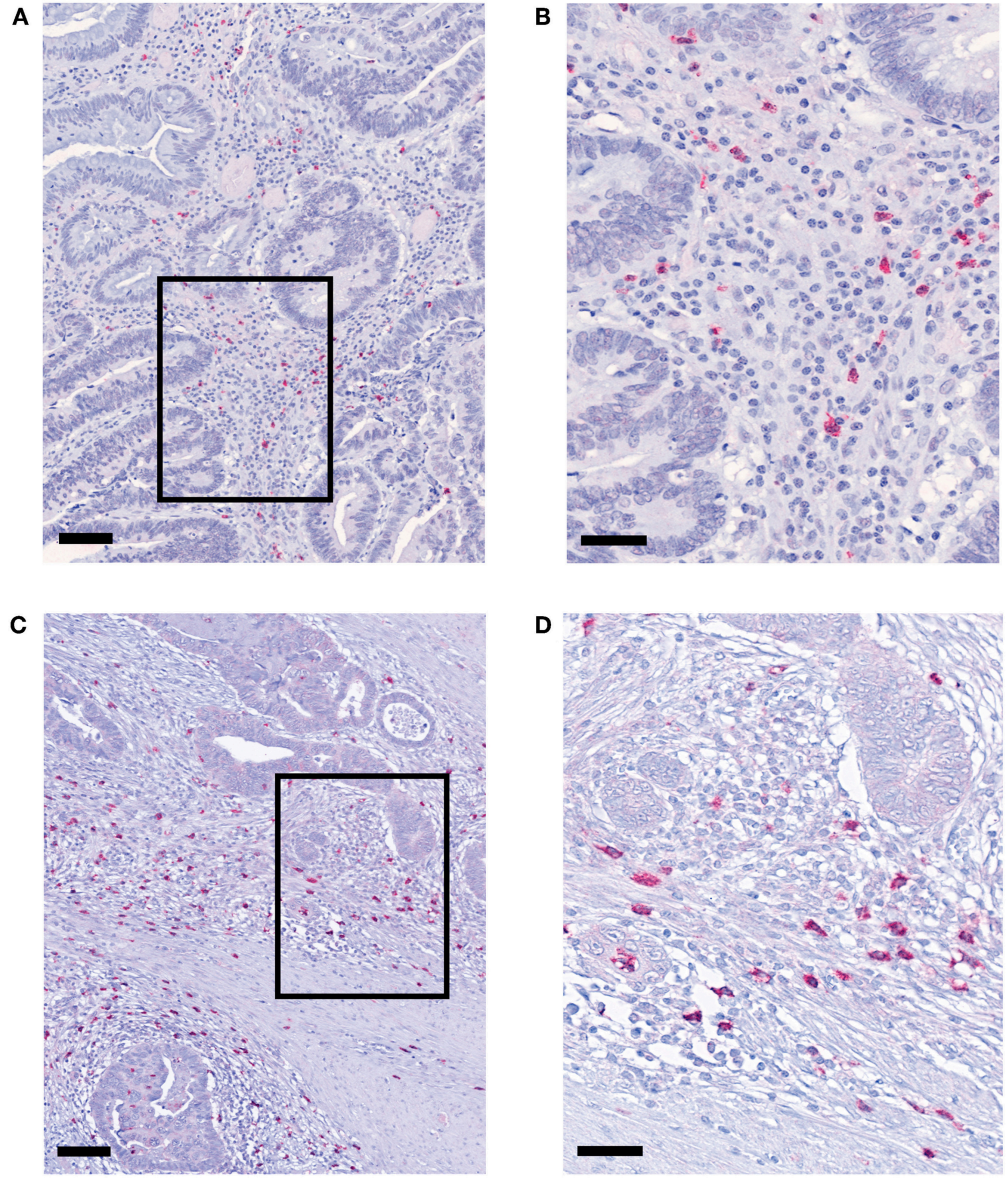
pDCs are abundant in tissues from non-treated and nRCT-treated rectal cancer patients. Immunohistochemical stainings were performed to detect pDCs in tissue specimens from non-matched untreated (*n* = 28) and nRCT-treated (*n* = 40) rectal cancer patients. As representative examples, the presence of infiltrating pDCs in a histologically confirmed, **(A,B)** untreated and **(C,D)** treated rectal cancer tissue is demonstrated. Scale bars are **(A,C)** 100 μm or **(B,D)** 50 μm, respectively.

**Figure 2:**
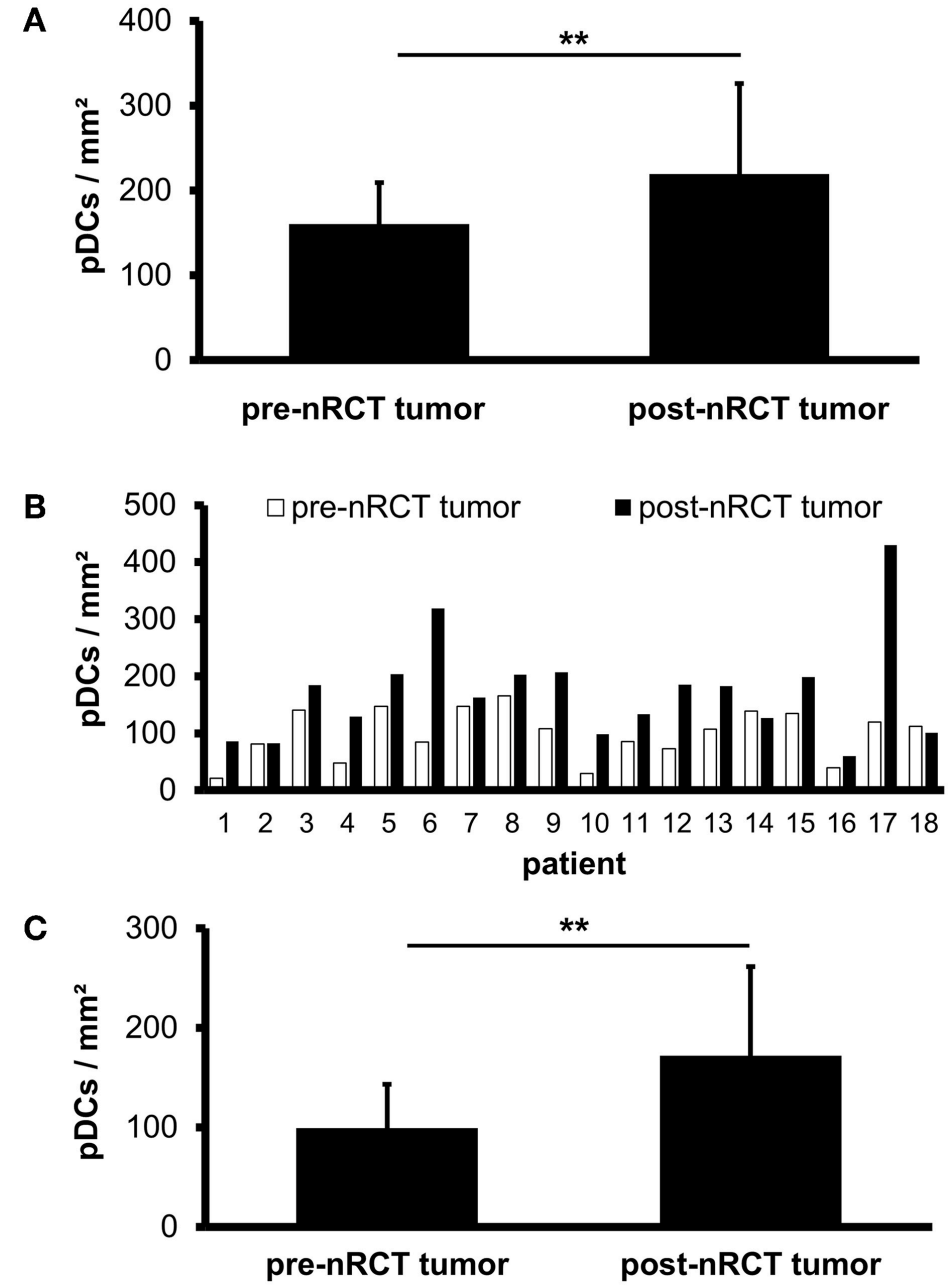
pDC infiltration is significantly higher in nRCT-treated rectal cancer tissues. **(A)** Immunohistochemical stainings were performed to evaluate the frequency of infiltrating pDCs in tissue specimens from non-matched, untreated and nRCT-treated rectal cancer patients. **(B,C)** In addition, pDC density in matched pre-nRCT or post-nRCT tumor specimens from 18 rectal cancer patients was analyzed. **(A)** The number of pDCs in non-matched untreated (*n* = 28) compared to nRCT-treated (*n* = 40) rectal cancer tissues is depicted. The results are presented as mean value ± SD of rectal cancer-infiltrating pDCs. Asterisks indicate a statistically significant difference (**p < 0.01). **(B)** pDC number in 18 matched pre-nRCT or post-nRCT tumor specimens is depicted for each patient. **(C)** pDC frequency in 18 matched pre-nRCT or post-nRCT tumor specimens is presented as mean value ± SD. Asterisks indicate a statistically significant difference (**p < 0.01).

pDCs essentially contribute to the regulation of innate and adaptive immunity and may play an important role in the immune defense against tumors. Recently, it has been demonstrated that pDCs are present in a variety of primary human tumor entities (26–28). However, little is known about the impact of nRCT on the density of tumor-infiltrating pDCs. Here, we address this issue by analyzing the frequency of pDCs in paraffin-embedded tissue specimens from non-treated (*n* = 28) and nRCT-treated (*n* = 40) rectal cancer patients (non-matched) with different clinicopathological characteristics (Table 1). BDCA-2^+^ pDCs were present in all non-treated and nRCT-treated rectal cancer tissue specimens (Figures 1A–D and data not shown) at varying frequencies. pDCs were preferentially located in the tumor stroma, where they were not equally distributed. Whereas some regions are characterized by an accumulation of pDCs (Figures 1A–D), other regions contained only low pDC numbers. Interestingly, the number of pDCs was significantly higher in the nRCT-treated cohort (219.3 ± 106.6 pDCs/mm^2^) than in the untreated cohort (160.3 ± 48.7 pDCs/mm^2^) as depicted in Figure 2A. In further experiments, matched pre-nRCT and post-nRCT tumor specimens from 18 rectal cancer patients were analyzed. pDC infiltration increased >10% after nRCT in 15 out of 18 patients (Figure 2B). As shown in Figure 2C, the frequency of pDCs was significantly higher in the nRCT-treated cohort (172.0 ± 89.6 pDCs/mm^2^) in comparison to the untreated cohort (99.2 ± 44.1 pDCs/mm^2^), confirming the results obtained with the non-matched cohorts.

### nRCT Does Not Significantly Modulate the Frequency of Rectal Cancer-Infiltrating slanMo

**Figure 3:**
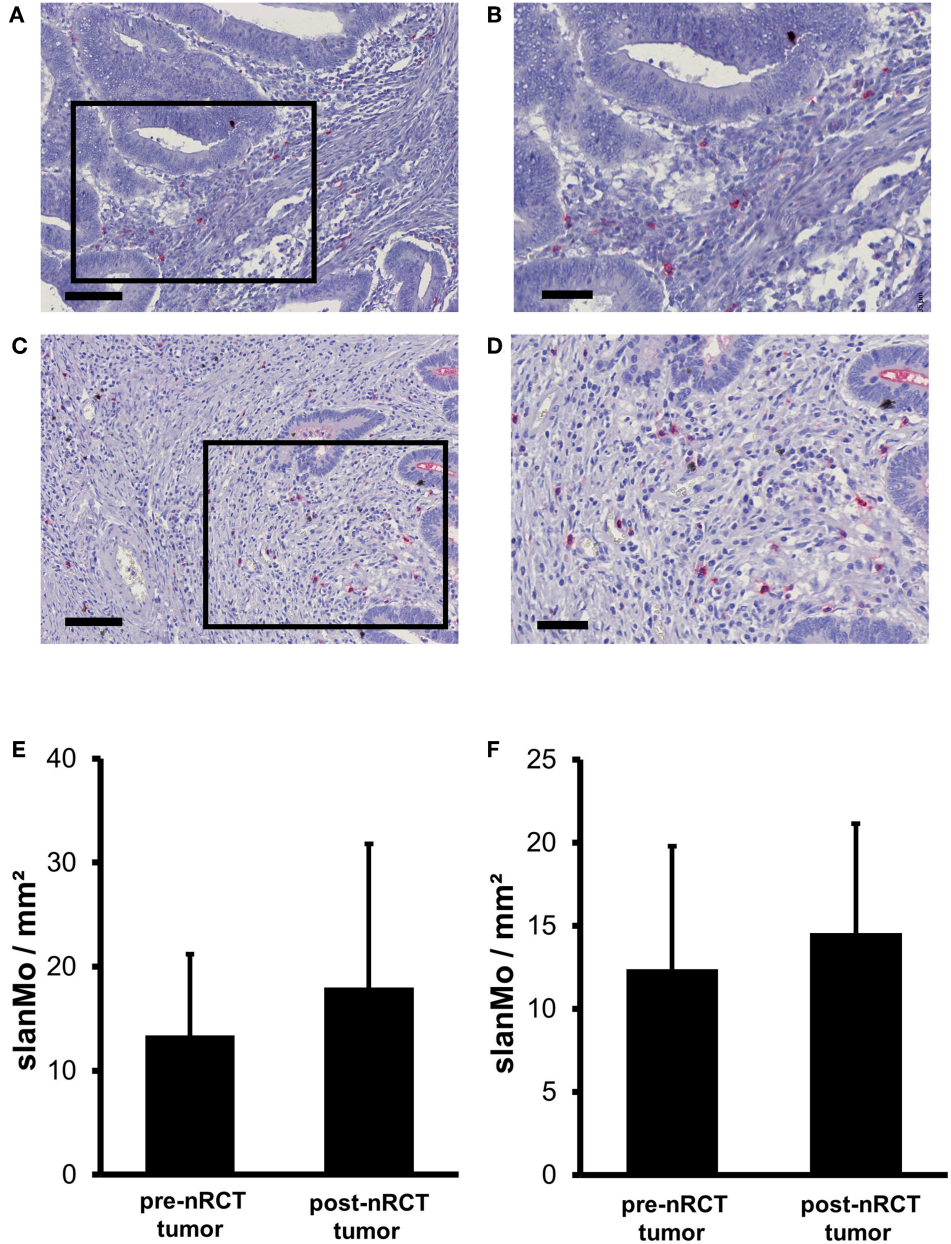
nRCT does not significantly alter slanMo frequency in rectal cancer tissues. **(A–F)** Immunohistochemical stainings were conducted to determine the density of infiltrating slanMo in tissue specimens from non-matched and matched untreated and nRCT-treated rectal cancer patients. **(A–D)** As representative examples, the presence of infiltrating slanMo in an **(A,B)** untreated and **(C,D)** treated rectal cancer tissue is shown. Scale bars are **(A,C)** 100 μm or **(B,D)** 50 μm, respectively. **(E)** The number of slanMo in non-matched untreated (*n* = 28) compared to nRCT-treated (*n* = 40) rectal cancer tissues is depicted. The results are presented as mean value ± SD of rectal cancer-infiltrating slanMo. **(F)** The frequency of slanMo in 20 matched pre-nRCT or post-nRCT tumor specimens is demonstrated. The results are presented as mean value ± SD of rectal cancer-infiltrating slanMo.

Previous studies have demonstrated that slanMo accumulate in primary tumor tissues of clear cell renal cell carcinoma (ccRCC) patients and in metastatic lymph nodes from cancer patients (42, 43). In the present study, we investigated whether infiltrating slanMo are detectable in rectal cancer tissues and whether nRCT can modulate their frequency. When analyzing the tissue specimens from non-treated and nRCT-treated rectal cancer patients (non-matched), we found that slanMo are present in 27 out of 28 non-treated and in all 40 nRCT-treated tissues (Figures 3A–D and data not shown) at varying frequencies. slanMo were preferentially located in the tumor stroma. In contrast to pDCs, nRCT only slightly increased the density of slanMo in the treated cohort (18 ± 13.8 slanMo/mm^2^) compared to the untreated (13.4 ± 7.8 slanMo/mm^2^) cohort (Figure 3E). These findings were confirmed when evaluating matched pre-nRCT and post-nRCT tumor specimens from 20 rectal cancer patients. Again, nRCT did not significantly modulate the frequency of rectal cancer-infiltrating slanMo (pre-nRCT: 12.4 ± 7.4 slanMo/mm^2^, post-nRCT: 14.6 ± 6.6 slanMo/mm^2^) as depicted in Figure 3F.

### nRCT Significantly Enhances the Proportion of GrzB-Expressing CD8^+^ T Cells in Rectal Cancer

**Figure 4:**
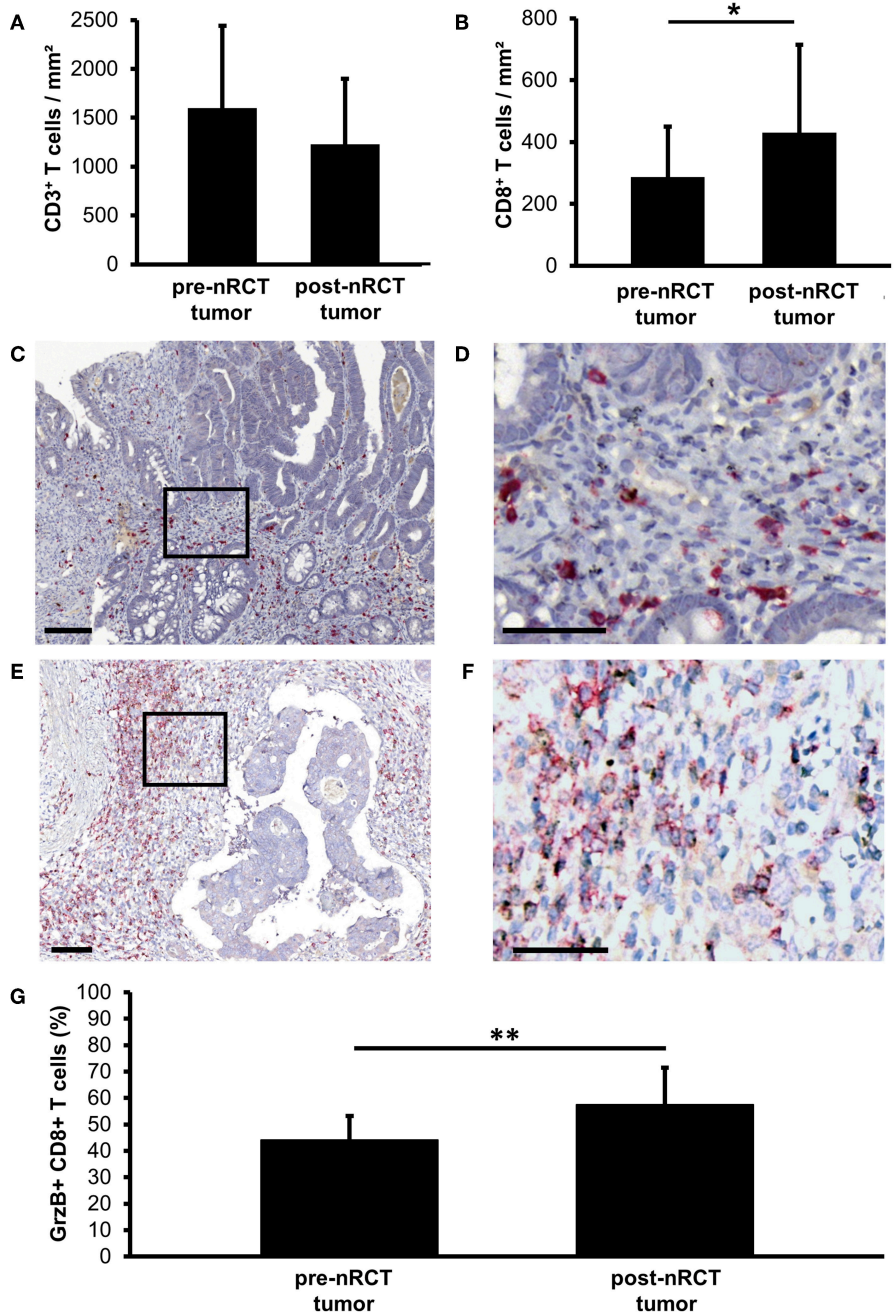
nRCT significantly increases the proportion of rectal cancer-infiltrating GrzB^+^ CD8^+^ T cells. **(A–G)** Immunohistochemical stainings were performed to explore the frequency of rectal cancer-infiltrating CD3^+^ T lymphocytes, total CD8^+^ T lymphocytes, and GrzB^+^ CD8^+^ T cells in matched pre-nRCT and post-nRCT tumor samples. The frequency of **(A)** CD3^+^ cells and **(B)** CD8^+^ T cells in 18 matched pre-nRCT or post-nRCT tumor specimens is presented as mean value ± SD. Asterisks indicate a statistically significant difference (*p < 0.05). **(C–F)** As representative examples, the presence of infiltrating GrzB^+^ CD8^+^ T cells in an **(C,D)** untreated and **(E,F)** nRCT-treated rectal cancer tissue is demonstrated. Scale bars are **(C,E)** 100 μm or **(D,F)** 50 μm, respectively. **(G)** The percentage of GrzB-expressing CD8^+^ T cells in 18 matched pre-nRCT or post-nRCT tumor specimens is presented as mean value ± SD. Asterisks indicate a statistically significant difference (**p < 0.01).

**Figure 5:**
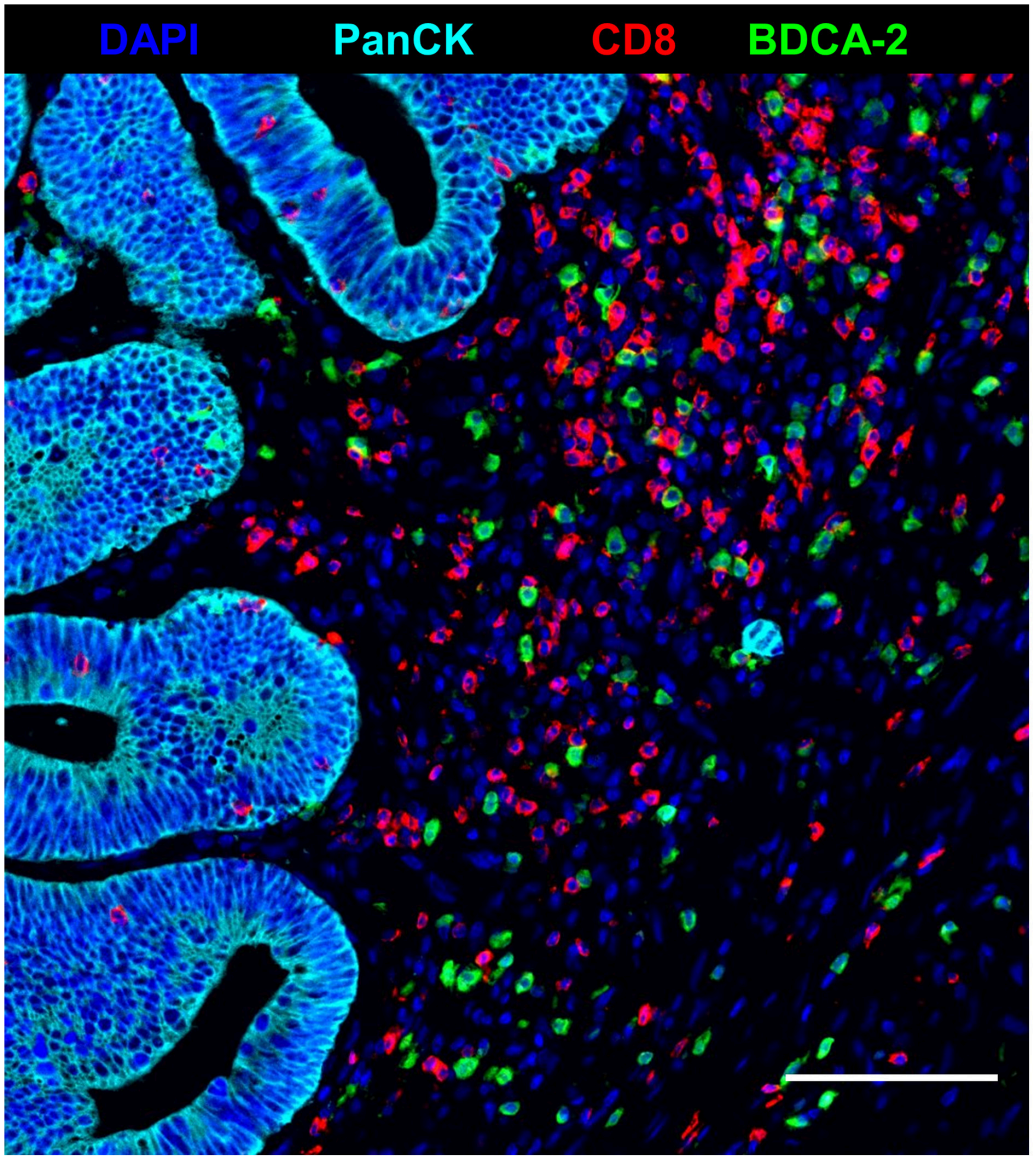
Co-localization of pDC and CD8^+^ T cells in nRCT-treated rectal cancer tissues. Immunofluorescence multiplex staining was performed to detect a co-localization of pDC and CD8^+^ T cells in four post-nRCT tumor samples. As a representative example, an image of a tissue region containing high numbers of rectal cancer-infiltrating pDC and CD8^+^ T cells is shown. Scale bar is 100 μm.

Recent studies have demonstrated that tumor-infiltrating T cells play an important role for the clinical outcome of colorectal cancer patients (2–5). Based on these findings, we explored the impact of nRCT on the frequency of rectal cancer-infiltrating CD3^+^ T lymphocytes, total CD8^+^ T lymphocytes, and GrzB-expressing CD8^+^ T cells in matched pre-nRCT and post-nRCT tumor samples of 18 patients. As demonstrated in Figure 4A, the number of rectal cancer-infiltrating CD3^+^ T cells was not significantly altered by nRCT (pre-nRCT: 1598.1 ± 842.4 CD3^+^ T cells/mm^2^, post-nRCT: 1228.8 ± 671.6 CD3^+^ T cells/mm^2^). In contrast, nRCT significantly increased the frequency of total CD8^+^ T cells in the nRCT-treated cohort (429.9 ± 284.2 CD8^+^ T cells/mm^2^) compared to the untreated cohort (286.8 ± 162.6 CD8^+^ T cells/mm^2^) as depicted in Figure 4B. The number of the CD8^+^ T cells was increased in 14 out of 18 post-nRCT tumor samples (data not shown). In 12 out of these 14 tissues, a simultaneous accumulation of CD8^+^ T cells and pDCs was detected. Within the total rectal cancer-infiltrating CD8^+^ T cell compartment, CD8^+^ T lymphocytes expressing the cytotoxic effector molecule GrzB were also detectable (Figures 4C–F). Notably, the percentage of GrzB^+^ CD8^+^ T cells was significantly higher in the nRCT-treated cohort (57.5 ± 14.4% GrzB^+^ CD8^+^ T cells) in comparison to the untreated cohort (44.2 ± 9.4% GrzB^+^ CD8^+^ T cells) as shown in Figure 4G. Following these findings, we explored whether CD8^+^ T cells co-localize with pDCs in four nRCT-treated rectal cancer tissues. As demonstrated in Figure 5, regions containing a high density of pDC and CD8^+^ T cells were detectable in all analyzed rectal cancer tissues.

### nRCT Increases the Proportion of iNOS- or TNF-α-Expressing slanMo in Rectal Cancer

**Figure 6:**
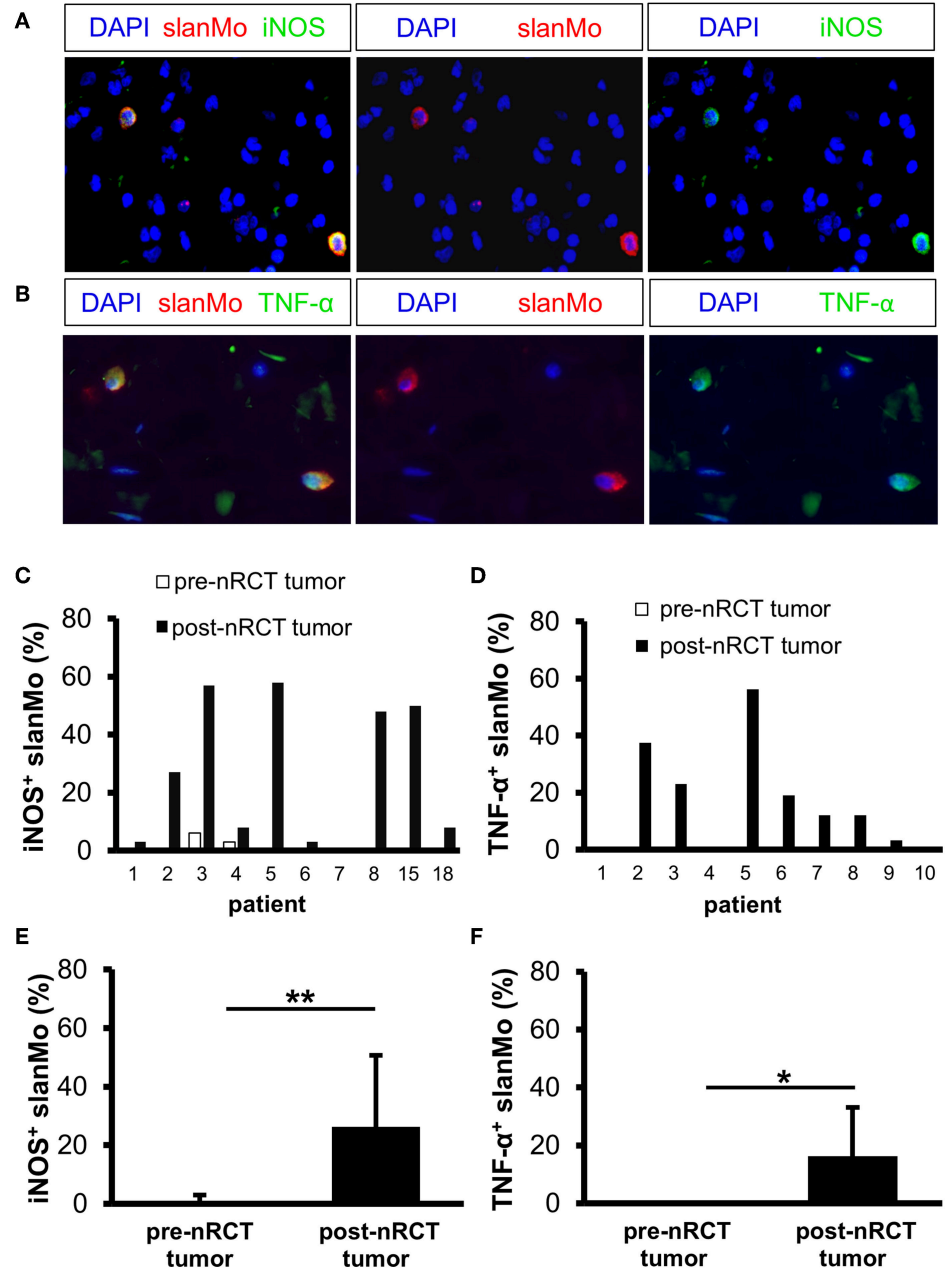
nRCT significantly increases the proportion of iNOS- and TNF-α-producing slanMo in rectal cancer tissues. **(A–F)** Immunofluorescence stainings were performed to evaluate the presence and percentage of iNOS- and TNF-α-expressing slanMo in matched pre-nRCT or post-nRCT tumor specimens from 10 rectal cancer patients. **(A,B)** As representative examples, images of **(A)** single iNOS or slan and **(B)** single TNF-α or slan stainings as well as merged images are depicted. Original magnification was x400. **(C,D)** Percentage of **(C)** iNOS- and **(D)** TNF-α-expressing slanMo in matched pre-nRCT or post-nRCT tumor specimens is shown for each patient. The results are presented as mean value ± SD of the proportion of **(E)** iNOS- or **(F)** TNF-α-expressing slanMo in matched pre-nRCT or post-nRCT tumor specimens. Asterisks indicate a statistically significant difference (*p < 0.05, **p < 0.01).

iNOS and TNF-α were shown to mediate either tumor-promoting or antitumor effects and may therefore influence the efficacy of nRCT in cancer patients (44, 45). Following our previous findings that iNOS- and/or TNF-α-expressing slanMo are detectable in tissue specimens of patients with various inflammatory disorders (35, 36, 46), we investigated whether nRCT modulates the percentage of iNOS- or TNF-α-producing slanMo in matched pre-nRCT and post-nRCT tumor specimens of 10 patients. While iNOS^+^ slanMo were present in 9 out of 10 post-nRCT rectal cancer tissues, TNF-α^+^ slanMo were detectable in 7 out of 10 tumor tissues obtained after treatment at varying percentages (Figures 6A–D). In contrast, iNOS^+^ slanMo were only found in 2 out of 10 pre-nRCT rectal cancer tissues, while TNF-α^+^ slanMo were absent in these tissues (Figures 6C,D). As depicted in Figure 6E, the proportion of iNOS^+^ slanMo was significantly higher in post-nRCT tumor tissues (26 ± 24% iNOS^+^ slanMo) compared to pre-nRCT tumor specimen (1 ± 2% iNOS^+^ slanMo). In addition, nRCT significantly increased the percentage of slanMo locally expressing TNF-α in rectal cancer tissues (Figure 6F).

### nRCT Significantly Increases the Proportion of CD83^+^ pDCs in Rectal Cancer

**Figure 7:**
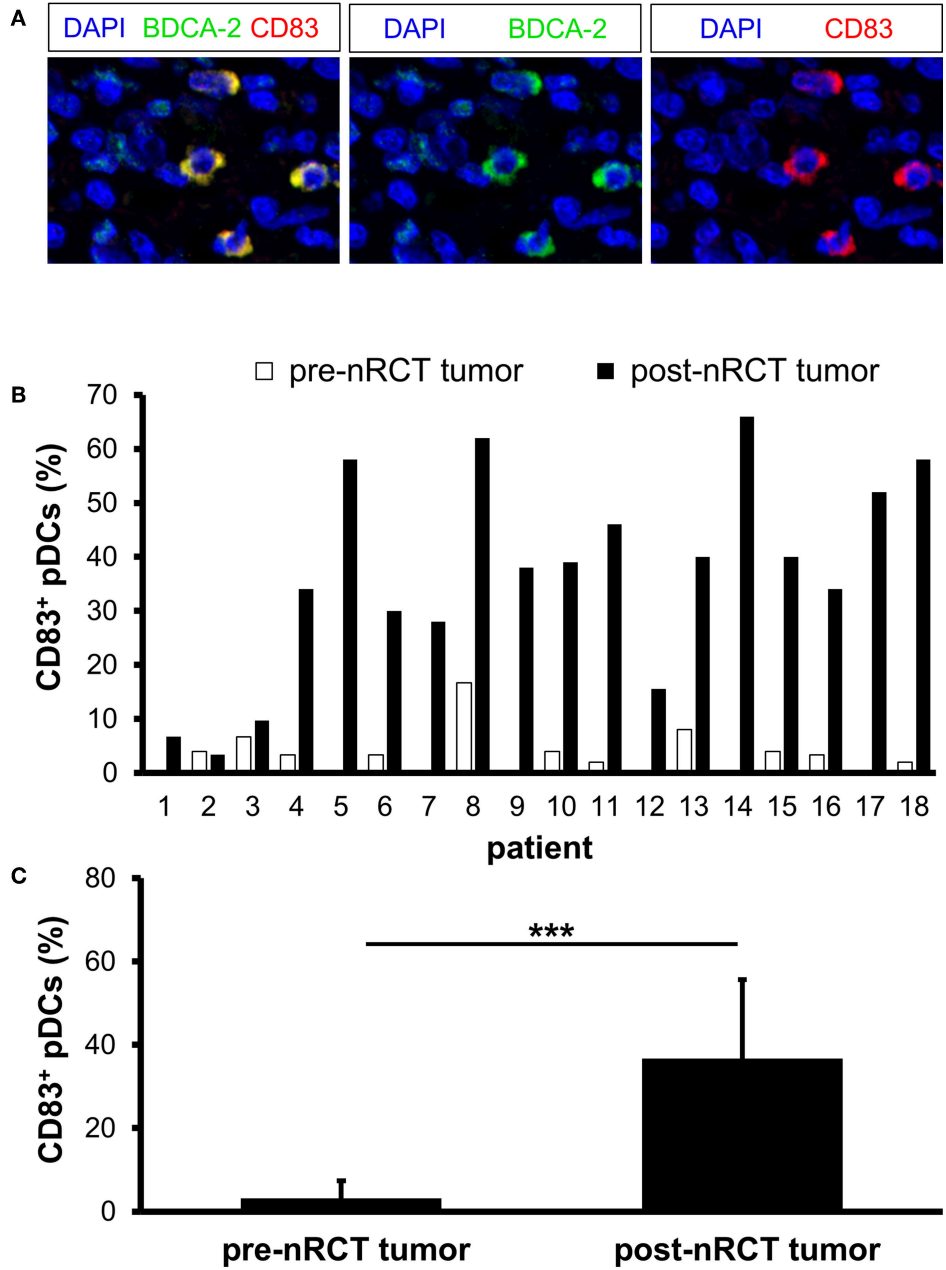
nRCT increases the proportion of mature pDCs in rectal cancer tissues. **(A–C)** Immunofluorescence stainings were conducted to analyze the presence and percentage of CD83-expressing pDCs in matched pre-nRCT or post-nRCT tumor specimens from 18 rectal cancer patients. **(A)** As representative example, images of single CD83 or BDCA-2 stainings as well as merged images are depicted. Original magnification was x400. **(B)** Percentage of CD83-expressing pDCs in matched pre-nRCT or post-nRCT tumor specimens is depicted for each patient. **(C)** The results are presented as mean value ± SD of the percentage of CD83^+^ pDCs in matched pre-nRCT or post-nRCT tumor specimens. Asterisks indicate a statistically significant difference (***p < 0.001).

To investigate whether nRCT influences the maturation status of rectal cancer-infiltrating pDCs, we analyzed the proportion of pDCs expressing the maturation marker CD83 in matched pre-nRCT and post-nRCT tumor samples of 18 patients. CD83^+^ pDCs were detectable in all post-nRCT rectal cancer tissues, but only in 11 out of 18 tumor tissues before nRCT at varying percentages (Figures 7A,B). In 13 post-nRCT rectal cancer tissues, ≥30% of pDCs expressing CD83 were present, providing evidence that these tissues can contain a marked proportion of mature pDCs (Figure 7B). As shown in Figure 7C, the percentage of CD83^+^ pDCs was significantly higher in post-nRCT tumor tissues (36.7 ± 18.9% CD83^+^ pDCs) in comparison to pre-nRCT tumor specimens (3.2 ± 4.2% CD83^+^ pDCs), indicating that nRCT can profoundly enhance the proportion of mature pDCs in rectal cancer tissues.

### nRCT Significantly Enhances the Percentage of IFN-α-Expressing pDCs in Rectal Cancer

**Figure 8:**
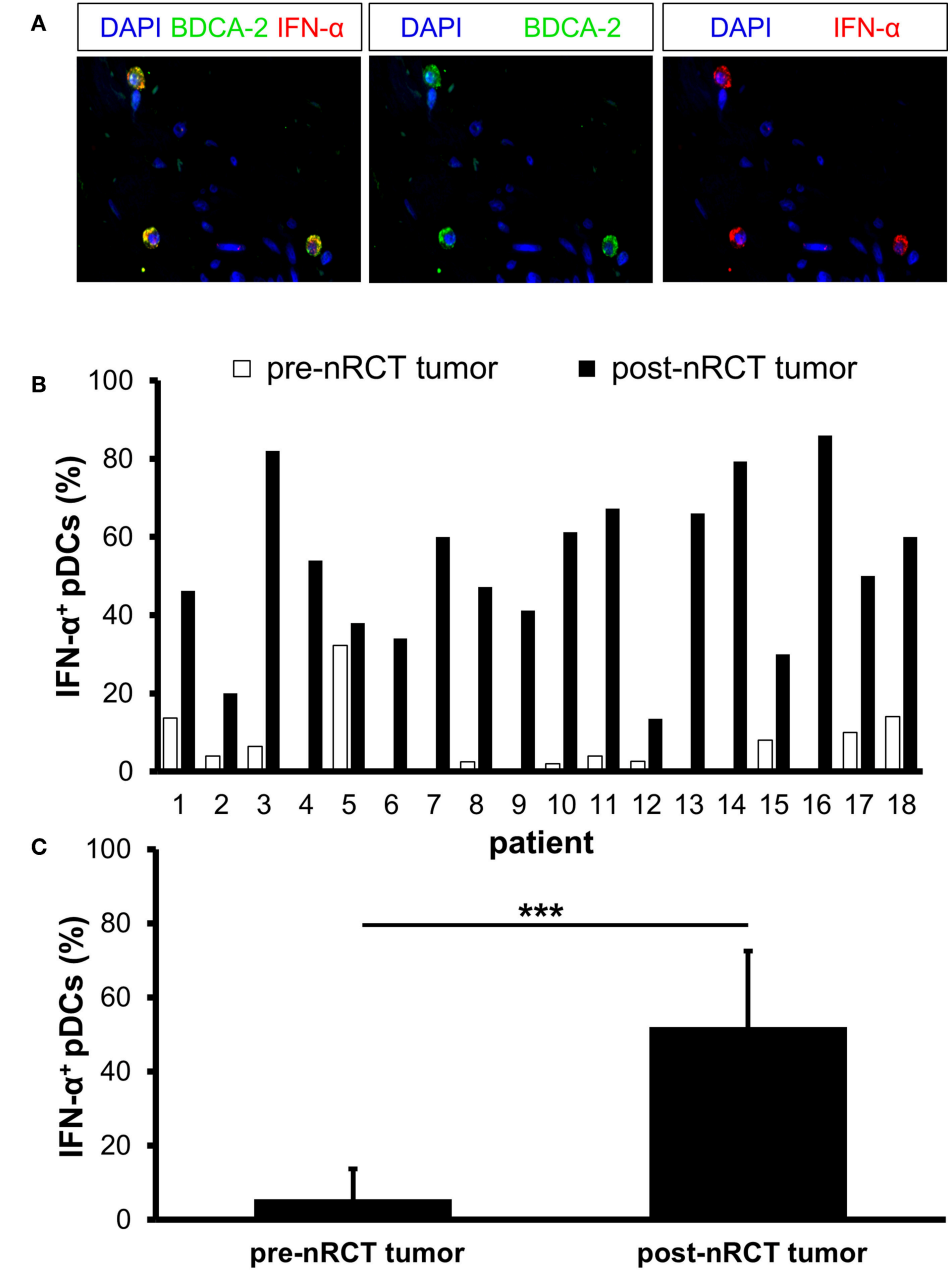
nRCT significantly enhances the percentage of IFN-α-expressing pDCs in rectal cancer tissues. **(A–C)** Immunofluorescence stainings were performed to determine the presence and percentage of IFN-α-expressing pDCs in matched pre-nRCT or post-nRCT tumor specimens from 18 rectal cancer patients. **(A)** As representative example, images of single IFN-α or BDCA-2 stainings as well as merged images are shown. Original magnification was x400. **(B)** Percentage of IFN-α-expressing pDCs in matched pre-nRCT or post-nRCT tumor specimens is depicted for each patient. **(C)** The results are presented as mean value ± SD of the proportion of IFN-α^+^ pDCs in matched pre-nRCT or post-nRCT tumor specimens. Asterisks indicate a statistically significant difference (***p < 0.001).

Activated pDCs are major producers of IFN-α, which may essentially contribute to the antitumor effects mediated by radio- and chemotherapy (18, 19). Following these findings, we determined the impact of nRCT on the proportion of IFN-α-expressing pDCs in matched pre-nRCT and post-nRCT tumor samples of 18 patients. Whereas, IFN-α^+^ pDCs were only present in 11 out of 18 pre-nRCT rectal cancer tissues, infiltrating pDCs locally expressing IFN-α were found in all post-nRCT tumor samples at varying percentages (Figures 8A,B). In 16 post-nRCT rectal cancer tissues, ≥30% of IFN-α^+^ pDCs were detectable (Figure 8B). The percentage of IFN-α^+^ pDCs was significantly higher in post-nRCT tumor tissues (52.0 ± 20.5% IFN-α^+^ pDCs) compared to pre-nRCT tumor specimen (5.5 ± 8.1% IFN-α^+^ pDCs) as depicted in Figure 8C. These results provide evidence that nRCT can profoundly increase the proportion of pDCs locally expressing IFN-α in rectal cancer tissues.

### Rectal Cancer-Infiltrating pDCs Express the Chemokines CXCL10 and CCL4

**Figure 9:**
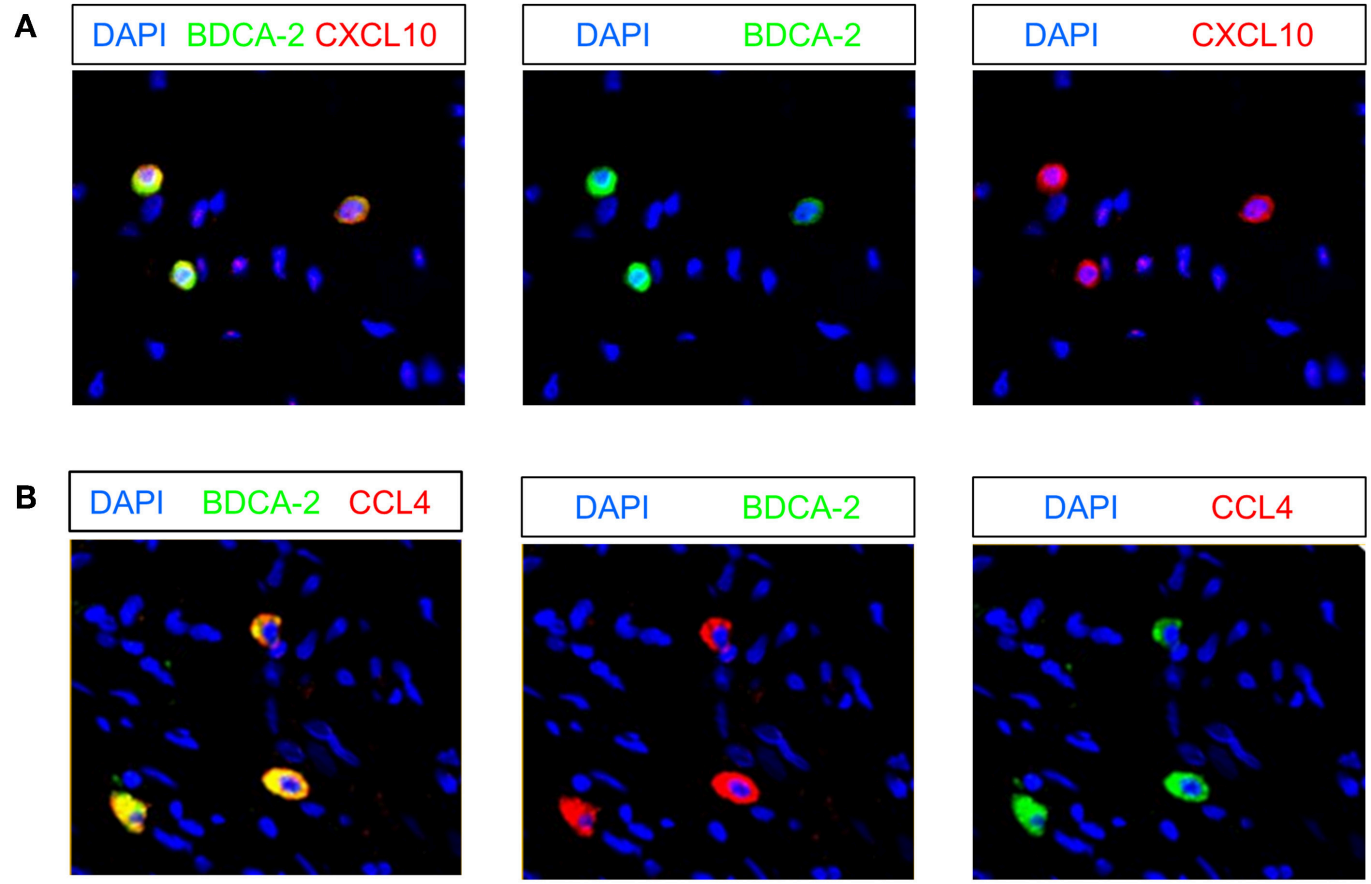
Rectal cancer-infiltrating pDCs can express CXCL10 and CCL4. **(A,B)** Immunofluorescence stainings were conducted to analyze the presence of CXCL10- or CCL4-expressing pDCs in 9 pre-nRCT and 18 post-nRCT tumor specimens of the matched cohort. As representative examples, images of single **(A)** CXCL10 and BDCA-2 or single **(B)** CCL4 and BDCA-2 stainings as well as merged images are shown. Original magnification was x200.

Recently, it has been shown that pDCs are able to produce various chemokines such as CCL4, CCL5, and CXCL10 (19, 47) that can promote the migration of T cells to tumor sites. Following these observations, we investigated the presence of pDCs expressing these chemokines in 9 pre-nRCT and 18 post-nRCT tumor specimens of the matched cohort. Whereas, CXCL10- or CCL4-expressing pDCs were not found in pre-nRCT tumor samples, CXCL10^+^ pDCs were detectable in 14 out of 18 and CCL4^+^ pDCs in 12 out of 18 post-nRCT tumor samples (Figures 9A,B). CCL5-expressing pDCs were not present in these tumor tissues (data not shown). These results imply that pDCs can contribute to the secretion of important chemokines for T-cell migration to rectal cancer tissues after nRCT.

## Discussion

Recent studies have revealed that DCs, as key regulators of innate and adaptive immunity, are a component of the immune architecture in colorectal cancer and may influence the clinical outcome of patients. When exploring the presence of human DCs in colorectal cancer, it has been reported that S-100^+^ DCs are detectable in almost all colorectal specimens and are mainly located in the tumor stroma (48–50). Increasing numbers of S-100^+^ DCs infiltrating tumor epithelium correlated with higher numbers of intraepithelial CD4^+^ and CD8^+^ T cells (49). Whereas, several studies provided evidence that a higher density of S-100^+^ DCs was associated with improved survival of patients (48, 51–53), other studies did not find a statistically significant correlation between S-100^+^ DC infiltration and a favorable clinical outcome (49, 50). In addition, it has been shown that DCs expressing the maturation marker CD83 are present in colorectal cancer (53–55). These DCs were found predominantly in the invasive margin, often in clusters with lymphocytes (55). Gulubova et al. demonstrated that patients with locally advanced tumors had significantly lower infiltration of CD83^+^ DCs in tumor stroma and in the invasive margin (53). Whereas all these findings are based on the detection of general marker molecules for DCs, studies investigating the presence of distinct human DC subsets in colon cancer tissues are rather limited. When exploring the density and distribution of pDCs in rectal cancer, we found that pDCs are present in all non-treated rectal cancer specimens at varying frequencies and are preferentially located in the tumor stroma. These results further substantiate recent studies, indicating that pDCs are detectable in colorectal cancer (51, 56). Previously, we and others have determined the presence of slanMo in primary tumor samples and derived metastases. Vermi et al. have found an accumulation of slanMo in metastatic lymph nodes from cancer patients (42). In addition, we have detected slanMo in the majority of primary tumor specimens, metastatic lymph nodes, and distant metastases from ccRCC patients (43). Further findings have revealed that ccRCC-infiltrating slanDCs display a tolerogenic phenotype and that higher slanDC numbers are associated with a reduced survival of ccRCC patients (43). More recently, it has been shown that slanMo are also present in bone marrow specimens of multiple myeloma patients and in various types of non-Hodgkin lymphomas (57, 58). In the present study, we observed that slanMo are detectable in almost all non-treated rectal cancer specimens at varying frequencies and are preferentially located in the tumor stroma. The slanMo frequency is markedly lower in comparison to rectal cancer-infiltrating pDCs, but higher compared to RCC-infiltrating slanMo (43). So far, only little is known about the impact of nRCT on the frequency of tumor-infiltrating DCs. When exploring the influence of nRCT on the number of infiltrating pDCs and slanMo in rectal cancer, we found a significantly higher number of pDCs in nRCT-treated tissue specimens, whereas the frequency of slanMo remained stable after nRCT.

CD4^+^ and CD8^+^ T lymphocytes are important effector cells of adaptive antitumor immunity. CD8^+^ effector T cells efficiently recognize and destroy tumor cells. CD4^+^ effector T cells enhance the ability of DCs to induce CD8^+^ T cell responses. They also provide help for the maintenance and expansion of CD8^+^ T cells and can eliminate tumor cells directly. In addition, CD4^+^ T cells are able to promote the differentiation of B cells into antibody-producing plasma cells. Based on these properties, tumor-infiltrating effector T cells efficiently influence tumor growth and play an essential role for the clinical outcome of colorectal cancer patients (2–5). Here, we explored the impact of nRCT on the frequency of rectal cancer-infiltrating T lymphocytes in matched pre-nRCT and post-nRCT tumor samples. We found that nRCT significantly increases the number of total CD8^+^ T cells as well as the percentage of CD8^+^ T cells expressing GrzB in the nRCT-treated cohort. These results are in agreement with previous studies, indicating that nRCT can significantly enhance the density of infiltrating CD8^+^ T cells in rectal cancer. Thus, it has been reported that the frequency of rectal cancer-infiltrating CD8^+^ T cells and the proportion of GrzB^+^ CD8^+^ T cells is markedly increased by nRCT (59–62). In addition, a high density of CD8^+^ T cells prior to treatment was associated with a good response to nRCT and was linked to a better clinical outcome (59–61). However, McCoy et al. did not observe an association between the frequency of rectal-cancer infiltrating CD8^+^ T cells prior to treatment and response to nRCT (63).

iNOS and TNF-α are important molecules that have an impact on carcinogenesis and cancer progression and may influence the clinical response of patients to various treatment modalities. Both molecules play a dual role in cancer by mediating tumor-promoting or antitumor effects (44, 45). Thus, iNOS and TNF-α were shown to promote tumor proliferation, angiogenesis, invasiveness, and metastasis. Further studies have revealed that these molecules can also efficiently impair tumor growth by various mechanisms such as the inhibition of proliferation, the induction of apoptosis, and the recruitment of tumor-reactive T cells (44, 45). Previously, we have identified slanMo as inflammatory, iNOS- and/or TNF-α-expressing cells in tissues specimens of patients with psoriasis, lupus erythematosus, or graft-vs.-host disease (35, 36, 46). Here, we determined the impact of nRCT on the percentage of iNOS- or TNF-α-expressing slanMo in matched pre-nRCT and post-nRCT rectal cancer specimens. iNOS^+^ or TNF-α^+^ slanMo were rare or absent in pre-nRCT tissues. However, nRCT significantly augmented the proportion of infiltrating slanMo locally expressing iNOS- or TNF-α in rectal cancer. The nRCT-mediated increase of iNOS-producing slanMo is in line with a recent study, demonstrating that low dose irradiation induces iNOS expression in melanoma-infiltrating mouse macrophages, resulting in an enhanced recruitment of T cells (64). In addition, Klug et al. observed that low dose irradiation leads to an accumulation of iNOS^+^ macrophages and intraepithelial T cells in tissue specimens of pancreatic cancer patients (64).

Type I IFN play a key role in antitumor immunity (65). They promote the maturation and antigen-presenting capacity of DCs as well as their migration to lymph nodes. Furthermore, type I IFN stimulate the release of proinflammatory cytokines by macrophages and improve the tumor-directed cytotoxic activity mediated by CD8^+^ T cells and NK cells. Accumulating evidence indicates that type I IFN can essentially contribute to the beneficial effects mediated by chemotherapy and radiotherapy (9, 66). Thus, it has been demonstrated that the efficacy of anthracycline-based chemotherapy against established tumor in mice is critically dependent on type I IFN (66). Furthermore, it has been reported that radiotherapy increases the intratumoral type I IFN expression in mice (67). Type I IFN were shown to enhance the cross-priming and T-cell stimulatory capacity of tumor-infiltrating DCs leading to tumor regression (67). pDCs are major producers of type I IFN following stimulation with toll-like receptor 7 and 9 agonists (18, 19). However, recent studies have revealed that tumor-infiltrating pDCs are defective at producing type I IFN in response to toll-like receptor agonists (26, 28, 29). When analyzing matched pre-nRCT and post-nRCT rectal cancer specimens, we found that only a small proportion of IFN-α-expressing pDCs is detectable prior nRCT. Notably, nRCT profoundly increased the proportion of pDCs locally expressing IFN-α. Together with our finding that nRCT also enhances the percentage of CD83^+^ pDCs, these results reveal that nRCT promotes the maturation and IFN-α production of rectal cancer-infiltrating pDCs.

pDCs are capable of producing various chemokines such as CCL4, CCL5, and CXCL10, which attract T cells to sites of infection and inflammation (19, 47). In addition, it has been reported that these chemokines play an essential role for the trafficking of T cells to tumor tissues. Thus, it has been shown that the expression of CCL4, CCL5, and CXCL10 in melanoma metastases is associated with the recruitment of CD8^+^ T cells (68). Moreover, a recent study has revealed that CCL5 and CXCL10 expressed by colorectal cancer tissues promote the migration of GrzB^+^ CD8^+^ T cells (69). When investigating the expression of these chemokines by rectal cancer-infiltrating pDCs, we observed that CCL4- or CXCL10-expressing pDCs are present in the majority of post-nRCT tumor specimens, whereas they are absent in pre-nRCT tissue samples. These findings indicate that nRCT can induce CXCL10 and CCL4 expression in rectal cancer-infiltrating pDCs. In addition, they imply that pDCs can contribute to the production of important chemokines for T-cell migration to rectal cancer tissues after nRCT.

Taken together, we found that nRCT significantly increases the percentage of rectal cancer-infiltrating slanMo locally expressing iNOS and TNF-α, which can either mediate tumor-promoting or antitumor effects and may affect the efficacy of nRCT in rectal cancer patients. Moreover, we demonstrated for the first time that nRCT results in an accumulation of pDCs as well as an increased proportion of CD83- and IFN-α-expressing pDCs in rectal cancer. In addition, the density of infiltrating GrzB^+^ CD8^+^ T cells was significantly enhanced by nRCT. These findings indicate that nRCT can harness antitumor responses by converting immature, non-activated pDCs into mature, inflammatory cells and by increasing the frequency of CD8^+^ T cells expressing the cytotoxic effector molecule GrzB. Activated pDCs and GrzB^+^ CD8^+^ T cells may contribute to the beneficial effect of nRCT in rectal cancer patients.

## Ethics Statement

This study was carried out in accordance with the recommendations of the ethical committee at the Faculty of Medicine Carl Gustav Carus of the Technische Universität Dresden, Germany. All subjects gave written informed consent in accordance with the Declaration of Helsinki. The protocol was approved by the ethical committee at the Faculty of Medicine Carl Gustav Carus of the Technische Universität Dresden, Germany.

## Author Contributions

FeW, UH, FrW, and IP performed experiments, analyzed data, and wrote the manuscript. RW, MaK, KF, and MA performed experiments and analyzed data. US, AJ, and AT analyzed data and wrote the manuscript. AB, CR, JW, KS, ET, MeK, GF, MB, MPB, DA, and GB analyzed data and revised the article. MS designed research, interpreted the data, and wrote the manuscript.

### Conflict of Interest Statement

The authors declare that the research was conducted in the absence of any commercial or financial relationships that could be construed as a potential conflict of interest.
